# Limitations in virtual clinical skills education for medical students during COVID-19

**DOI:** 10.36834/cmej.70240

**Published:** 2020-12-07

**Authors:** Yaanu Jeyakumar, Deepanshu Sharma, Giovanna Sirianni, Joyce Nyhof-Young, Mirek Otremba, Fok-Han Leung

**Affiliations:** 1 Faculty of Medicine, University of Toronto, Ontario, Canada; 2 Department of Family and Community Medicine, University of Toronto, Ontario, Canada; 3 Sunnybrook Health Sciences Centre, Ontario, Canada; 4 Division of General Internal Medicine, Mount Sinai Hospital, Ontario, Canada

With the novel Coronavirus disease (COVID-19) being declared a pandemic and global emergency, extreme measures have been taken to curb the transmission of the virus. The mandate of physical distancing has forced most undergraduate medical and other health professions programs to move their entire preclinical curricula online and provide education virtually. Furthermore, with the current shortage of personal protective equipment for front-line healthcare workers and the fear that students may transmit the virus unknowingly, most medical students have been removed entirely from the clinical learning environment.

Before this pandemic, many undergraduate medical programs were already adopting a “flipped-classroom” model of education for preclinical learners, which may have aided in their quick conversion to a virtual curriculum during the pandemic. Courses rooted in a transmission format have transitioned well to this new virtual curriculum model. Even courses requiring facilitated discussion and clinical problem solving in small groups have fared well during the changeover due to technological advancements in remote conferencing platforms. Unfortunately, the clinical skills education program for preclinical learners has experienced serious challenges in this transition.

The move to a virtual platform has been accomplished by disseminating content through a combination of online modules and pre-recorded videos of physicians performing history and physical examinations with patients. The benefit of these current approaches to virtual clinical skills education is that the online modules can provide a detailed approach to complex history and physical examinations, while the pre-recorded videos allow the learner to visualize the same examinations. A virtual curriculum also allows the medical student to rewatch or re-read more complex concepts. However, these modules and videos were not intended to be used as standalone teaching activities; they were designed to supplement the in-person learning that occurs in a clinical setting. The physical presence of students in clinical settings allows them to actively engage with patients and learn clinical skills through both observation and execution. Working closely with real, volunteer, and standardized patients throughout undergraduate medical education is crucial to developing and refining essential skills.

During the SARS outbreak in 2003, various measures were taken to replace the learner experience with patients; these included e-modules, video vignettes, digital games, and both live and mannequin-based simulated patients.^1^ While such strategies can help minimize educational disruptions, clinical encounters are considerably more visual, interactive, and demonstrative, with opportunities for students to ask questions and garner performance feedback. One novel strategy that has not been explored extensively in medical education is broadcasting patient encounters to medical learners through wearable technologies in real-time; this approach may be able to replicate, at least in part, the experience of an in-person clinical encounter.

Few studies have explored the practicality and effectiveness of first-person or point-of-view (POV) filming with wearable technologies in medical education. To date, it is only an established approach in fields such as ethnography.^2^ A group of researchers in Britain explored POV filming as a tool to clinically train final year medical students.^3^ A faculty physician utilized a POV camera on a head strap and students were able to observe the physician participating in ward handover, documenting ward round entry, and making decisions about treatment and medical prescription. The two-hour interactive session was well-received by the students in terms of enjoyment, interactivity, and utility, while being resource-efficient. In addition, POV filming can readily facilitate learning for large cohorts of learners. In 2014, a team of consultant surgeons at Barts Health NHS Trust used Google Glass to broadcast the UK’s first global, live-streamed surgical teaching session to 13,000 surgical students and health care professionals.^4^ While these wearable technologies appear capable of providing rich field experiences in the absence of in-person clinical training, many challenges remain including cost, feasibility, patient privacy, confidentiality, consent, and data security. As research continues in this novel field, it may be possible to provide a set of best practices to avoid or minimize these issues.

It is unrealistic to expect to flawlessly replicate the lost experiences of in-person clinical skills training. However, an important goal of medical educators during unexpected clinical teaching restrictions should be to provide students with viable options to enhance their training. One promising approach to supplement the currently available virtual education for clinical skills training is the implementation of wearable camera-based technologies to facilitate POV learning. While there seem to be several advantages to this approach as discussed in this paper, a clear need exists for more research on its effectiveness in medical education and education in other health professions.
